# Population-based resistance of *Mycobacterium tuberculosis* isolates to pyrazinamide and fluoroquinolones: results from a multicountry surveillance project

**DOI:** 10.1016/S1473-3099(16)30190-6

**Published:** 2016-10

**Authors:** Matteo Zignol, Anna S Dean, Natavan Alikhanova, Sönke Andres, Andrea Maurizio Cabibbe, Daniela Maria Cirillo, Andrei Dadu, Andries Dreyer, Michèle Driesen, Christopher Gilpin, Rumina Hasan, Zahra Hasan, Sven Hoffner, Ashaque Husain, Alamdar Hussain, Nazir Ismail, Mostofa Kamal, Mikael Mansjö, Lindiwe Mvusi, Stefan Niemann, Shaheed V Omar, Ejaz Qadeer, Leen Rigouts, Sabine Ruesch-Gerdes, Marco Schito, Mehriban Seyfaddinova, Alena Skrahina, Sabira Tahseen, William A Wells, Ya Diul Mukadi, Michael Kimerling, Katherine Floyd, Karin Weyer, Mario C Raviglione

**Affiliations:** a Global Tuberculosis Programme, World Health Organization, Geneva, Switzerland; b Scientific Research Institute of Lung Diseases, Baku, Azerbaijan; c National and Supranational Reference Laboratory for Mycobacterium, Borstel, Germany; d IRCCS San Raffaele Scientific Institute, Milan, Italy; e Regional Office for Europe, World Health Organization, Copenhagen, Denmark; f National Institute for Communicable Diseases, Sandringham, South Africa; g Mycobacteriology Unit, Institute of Tropical Medicine, Antwerp, Belgium; h Department of Pathology and Laboratory Medicine, Aga Khan University, Karachi, Pakistan; i Department of Microbiology, Public Health Agency of Sweden, Solna, Sweden; j Department of Microbiology, Tumor and Cell Biology, Karolinska Institute, Stockholm, Sweden; k National Tuberculosis Control Programme, Dhaka, Bangladesh; l National Tuberculosis Reference Laboratory, National Tuberculosis Control Programme, Islamabad, Pakistan; m University of Pretoria, Pretoria, South Africa; n National Institute of Diseases of the Chest and Hospital, Dhaka, Bangladesh; o Department of Microbiology, Public Health Agency of Sweden, Solna, Sweden; p Tuberculosis Control and Management, National Department of Health, Pretoria, South Africa; q National Tuberculosis Control Programme, Ministry of National Health Services, Regulation and Coordination, Islamabad, Pakistan; r Biomedical Sciences, Antwerp University, Antwerp, Belgium; s Critical Path Institute, Tucson, AZ, USA; t Republican Research and Practical Centre for Pulmonology and Tuberculosis, Minsk, Belarus; u Bureau for Global Health, US Agency for International Development, Washington, DC, USA; v KNCV Tuberculosis Foundation, The Hague, Netherlands

## Abstract

**Background:**

Pyrazinamide and fluoroquinolones are essential antituberculosis drugs in new rifampicin-sparing regimens. However, little information about the extent of resistance to these drugs at the population level is available.

**Methods:**

In a molecular epidemiology analysis, we used population-based surveys from Azerbaijan, Bangladesh, Belarus, Pakistan, and South Africa to investigate resistance to pyrazinamide and fluoroquinolones among patients with tuberculosis. Resistance to pyrazinamide was assessed by gene sequencing with the detection of resistance-conferring mutations in the *pncA* gene, and susceptibility testing to fluoroquinolones was conducted using the MGIT system.

**Findings:**

Pyrazinamide resistance was assessed in 4972 patients. Levels of resistance varied substantially in the surveyed settings (3·0–42·1%). In all settings, pyrazinamide resistance was significantly associated with rifampicin resistance. Among 5015 patients who underwent susceptibility testing to fluoroquinolones, proportions of resistance ranged from 1·0–16·6% for ofloxacin, to 0·5–12·4% for levofloxacin, and 0·9–14·6% for moxifloxacin when tested at 0·5 μg/mL. High levels of ofloxacin resistance were detected in Pakistan. Resistance to moxifloxacin and gatifloxacin when tested at 2 μg/mL was low in all countries.

**Interpretation:**

Although pyrazinamide resistance was significantly associated with rifampicin resistance, this drug may still be effective in 19–63% of patients with rifampicin-resistant tuberculosis. Even though the high level of resistance to ofloxacin found in Pakistan is worrisome because it might be the expression of extensive and unregulated use of fluoroquinolones in some parts of Asia, the negligible levels of resistance to fourth-generation fluoroquinolones documented in all survey sites is an encouraging finding. Rational use of this class of antibiotics should therefore be ensured to preserve its effectiveness.

**Funding:**

Bill & Melinda Gates Foundation, United States Agency for International Development, Global Alliance for Tuberculosis Drug Development.

## Introduction

With 9·6 million new cases and 1·5 million deaths estimated in 2014, tuberculosis represents a major global health problem and ranks alongside HIV as a leading cause of infectious-disease-related deaths.^1^ Although global incidence has been falling slowly during the past decade, the number of people affected every year remains daunting. Among the most serious obstacles to successful prevention and treatment of tuberculosis are the inadequate identification of individuals with latent tuberculosis infection who are at highest risk of developing the disease,^2^ insufficient capacity of health systems to rapidly identify and diagnose all tuberculosis cases (especially those with drug resistance),^3^ inappropriate management of contacts of infectious cases, long duration of treatment (especially for drug-resistant tuberculosis),^4^ concurrent infection with HIV, and worldwide spread of *Mycobacterium tuberculosis* strains that are resistant to the most effective antituberculosis agents.

To accelerate global progress in the control of tuberculosis, new drugs and shorter, easily administered regimens are needed to treat all forms of tuberculosis, including multidrug-resistant and extensively drug-resistant tuberculosis.

The use of a fourth-generation fluoroquinolone (ie, moxifloxacin or gatifloxacin) to shorten the treatment of drug-susceptible tuberculosis to 4 months has been recently assessed in three separate large trials (OFLOTUB,^5^ REMoxTB,^6^ and RIFAQUIN^7^). Unfortunately, none of these trial findings showed non-inferiority compared with the WHO-recommended 6-month standard regimen for the treatment of tuberculosis.^8^

A few new antituberculosis drugs have undergone clinical evaluation over the past decade. These include bedaquiline (a diary quinoline) and delamanid (a nitroimidazole), which have been recently approved by national regulatory authorities and recommended by WHO^1^ for use in selected patients with multidrug-resistant tuberculosis. Additionally, pretomanid, another nitroimidazole, is under evaluation in short multidrug regimens for the treatment of drug-susceptible and drug-resistant tuberculosis.^9^

**Research in context**

**Evidence before this study**

The combination of pyrazinamide plus a fourth-generation fluoroquinolone (moxifloxacin or gatifloxacin) is considered essential in novel rifampicin-sparing regimens for the treatment of tuberculosis and in shorter regimens for the treatment of multidrug-resistant tuberculosis. Understanding the background prevalence at population level of resistance to these drugs is critical to assess the feasibility of introducing new and shorter regimens in tuberculosis control programmes and the need for drug-susceptibility testing to accompany the introduction of these new regimens. For the past 20 years, levels of resistance to the most powerful first-line antituberculosis drugs, rifampicin and isoniazid, have been monitored in more than 150 countries worldwide through routine surveillance or ad-hoc population-based surveys. Results of these studies are reported to WHO. Susceptibility testing to fluoroquinolones and pyrazinamide is not routinely performed on all tuberculosis cases as part of drug resistance surveillance efforts. Therefore, population-representative surveillance data on levels of resistance to pyrazinamide and fourth-generation fluoroquinolones (moxifloxacin or gatifloxacin) among all patients with tuberculosis do not exist at present. We searched MEDLINE (1966 to March 20, 2016) and Embase (1980 to March 20, 2016), using the terms “tuberculosis”, “drug”, “resistance”, and “surveillance”, with restriction to English, French, and Spanish results. We also searched the database of the WHO global project on antituberculosis drug resistance surveillance, containing results of all published and unpublished national population-based antituberculosis drug resistance surveys and surveillance conducted worldwide (1994 to March 20, 2016).

**Added value of this study**

This study presents the results of the first population-based surveys investigating levels of resistance to pyrazinamide, ofloxacin, levofloxacin, moxifloxacin, and gatifloxacin among patients with tuberculosis in countries with high burden of tuberculosis and multidrug-resistant tuberculosis. In routine surveillance and patient management, testing for resistance to these drugs is restricted to certain patient groups, such as those with rifampicin resistance or a history of previous treatment for tuberculosis. This dataset therefore provides essential insight into the background proportions of resistance to these drugs at population level and in particular among newly diagnosed tuberculosis cases. Our work offers insight into the feasibility of introducing new tuberculosis treatment regimens and strategies for drug-susceptibility testing in these settings.

**Implications of all the available evidence**

Our results have programmatic implications for both treatment strategies and investment opportunities in the development and implementation of new diagnostic technologies. This work shows that the presence of rifampicin resistance, which currently is easily identified thanks to the wide availability of new rapid molecular technology, should prompt attention to the possibility of the simultaneous presence of resistance to pyrazinamide and, in some settings, the earlier generation fluoroquinolones. At the same time, resistance to the latest generation fluoroquinolones at the clinical breakpoint is still uncommon. This is a crucial finding for the design of standard regimens in different settings, and for guidance to regimen developers and diagnostic manufacturers. Our findings support public health policy makers in prioritisation and introduction of new regimens and algorithms for drug-susceptibility testing, and call for a rethinking of surveillance needs to ensure that more and better data are available to understand levels of resistance to pyrazinamide and fluoroquinolones in different epidemiological settings and patient groups.

Although individual new drugs are important, there remains a programmatic need for effective and shorter regimens, particularly for drug-resistant tuberculosis.^10^, ^11^ Many of the potential new regimens being proposed or tested contain at least one or more of the existing antituberculosis drugs. Notably, the combination of pyrazinamide plus moxifloxacin or gatifloxacin is considered essential in novel rifampicin-sparing regimens for the treatment of tuberculosis^9^, ^12^ and in shorter regimens for the treatment of multidrug-resistant tuberculosis.^10^, ^11^

When resistance to an individual drug within a treatment regimen emerges, a reduction in treatment efficacy is usually the result. Additionally, there is greater potential for generating resistance to the remaining drugs in the regimen. Ideally, a new regimen would be introduced in a population that has little or no pre-existing resistance. Therefore, an understanding of the background prevalence of resistance to all drugs included in new regimens is needed to assess the feasibility of shorter regimens and the need for drug-susceptibility testing to accompany regimen introduction.^13^ Whereas levels of rifampicin and isoniazid resistance are routinely monitored in most tuberculosis-endemic countries, susceptibility testing to fluoroquinolones and pyrazinamide is not routinely performed as part of drug resistance surveillance.^14^ Therefore, little population-representative surveillance data about levels of resistance to these drugs are available.

In this Article, we present the results of the first population-based surveys investigating levels of resistance to pyrazinamide, ofloxacin, levofloxacin, moxifloxacin, and gatifloxacin among patients with tuberculosis in countries with a high burden of tuberculosis and multidrug-resistant tuberculosis. We also investigate levels of cross resistance among fluoroquinolones.

## Methods

### Study design and participants

Drug resistance surveys are specially designed studies to measure antituberculosis drug resistance among a representative sample of notified patients with pulmonary tuberculosis. Details about the design of drug resistance surveys are provided elsewhere.^14^ Data presented in this Article were gathered from isolates from patients with tuberculosis enrolled in national surveys in Azerbaijan (2013),^15^ Bangladesh (2011),^16^ Pakistan (2013),^17^ and in subnational surveys in Minsk city, Belarus (2010)^18^ and in Gauteng and KwaZulu Natal provinces of South Africa (2014).^19^ These countries were selected because they represent a variety of programmatic and epidemiological settings. They all have high tuberculosis burden, with tuberculosis incidence varying between 58 per 100 000 and 834 per 100 000 population and proportion of tuberculosis cases with rifampicin resistance ranging from 4·9% to 49·1% (appendix). In all of these countries, uncomplicated tuberculosis is treated in the public health sector using a standardised regimen recommended by WHO.^8^ In Bangladesh and Pakistan in particular, a substantial number of patients are treated by private practitioners with variable drug combinations and schemes.

### Procedures

Sputum samples collected from patients enrolled in these surveys underwent culture and susceptibility testing to first-line antituberculosis drugs at the National tuberculosis Reference Laboratory using either the LJ proportion method (Azerbaijan, Bangladesh, Pakistan) or MGIT 960 (Becton Dickinson, Sparks, MD, USA; Belarus and South Africa). Isolates were then sent to selected WHO tuberculosis supranational reference laboratories where testing for resistance to pyrazinamide, ofloxacin, levofloxacin, moxifloxacin, and gatifloxacin was performed. Laboratory methods were standardised and all laboratories successfully passed proficiency testing for pyrazinamide and fluoroquinolones before starting the project. Resistance to pyrazinamide was assessed by sequencing for the detection of resistance-conferring mutations in the *pncA* gene (Rv2043c) and the promoter, located in the Rv2044c–Rv2043c intergenic region. The technologies employed were those already in use at the supranational reference laboratories and included Sanger sequencing using 3730xl (Thermo Fisher Scientific, MA, USA), next generation sequencing using Hiseq 2500 and MiSeq platforms (Illumina, San Diego, CA, USA), and Ion Torrent PGM (Thermo Fisher Scientific), according to manufacturers' instructions. The role of mutations in conferring resistance was assigned on the basis of available scientific literature^20^, ^21^ and online databases.^22^, ^23^, ^24^ Additionally, phenotypic susceptibility testing to pyrazinamide was conducted on MGIT 960 at the concentration of 100·0 μg/mL using the MGIT-PZA kit^25^ on all isolates with previously unreported mutations. The role of these mutations in conferring resistance was assigned according to the observed phenotype.

Susceptibility testing to fluoroquinolones was conducted using the MGIT system and stock solutions of drugs prepared in house. All isolates were tested for susceptibility to ofloxacin at 2·0 μg/mL and moxifloxacin at 0·5 μg/mL. Any isolate found to be resistant to either ofloxacin at 2·0 μg/mL or moxifloxacin at 0·5 μg/mL was subsequently tested for resistance to levofloxacin at 1·5 μg/mL, and moxifloxacin and gatifloxacin at 2·0 μg/mL. Moxifloxacin was tested at two concentrations because 0·5 μg/mL is considered the epidemiological cutoff and 2·0 μg/mL is considered the clinical breakpoint,^25^ defined as the concentration of an antibiotic that can be achieved in body fluids or target sites during optimal therapy. Isolates found to be susceptible to both ofloxacin at 2·0 μg/mL and moxifloxacin at 0·5 μg/mL were considered to be susceptible to levofloxacin at 1·5 μg/mL, and moxifloxacin and gatifloxacin at 2·0 μg/mL.

### Statistical analysis

We analysed data using version 12.0 of the Stata package, stratified for new cases, previously treated cases, all cases combined, cases of rifampicin-susceptible tuberculosis, and cases of rifampicin-resistant tuberculosis to investigate the association of resistance with treatment history and rifampicin resistance status. Due to the failure to regrow and test all strains, multiple imputation of missing values was performed across all settings for all drugs. Final imputation models for each drug included the following variables: age, sex, history of previous treatment, and rifampicin resistance status. Proportions of resistance within each group and 95% CIs were calculated by logistic regression, specifying robust standard errors to account for the cluster-based survey design in some countries (Bangladesh, Pakistan, South Africa). For drugs without any resistant cases, 95% CIs were calculated using the normal approximation to the binomial.

### Role of the funding source

The funders of the study had no role in study design, data collection, data analysis, data interpretation, or writing of the report. The corresponding author had full access to all the data in the study and had final responsibility for the decision to submit for publication.

## Results

In this retrospective study, not all isolates collected during the original surveys could be successfully regrown. The proportions of the original survey strains that could not be retrieved due to poor growth varied across countries: 5% (41/789) in Azerbaijan, 29% (389/1344) in Bangladesh, 10% (23/224) in Belarus, 6% (89/1592) in Pakistan, and 6% (52/929) in Gauteng province and 9% (65/756) in KwaZulu Natal province in South Africa. Individuals whose sample could not be re-grown did not differ from those with viable samples in relation to age, sex, history of treatment or rifampicin resistance status (data not shown).

Proportions of resistance to pyrazinamide by setting, history of previous treatment and rifampicin susceptibility are reported in table 1.

A total of 4972 patients were tested to investigate resistance to pyrazinamide in the five countries. Levels of resistance among all tuberculosis cases varied substantially (3·0–42·1% in the surveyed settings) and were lowest in Bangladesh, Pakistan, and South Africa. In all countries and for all patient groups, levels of resistance to pyrazinamide did not statistically differ from the levels of resistance to rifampicin (appendix); the only exception was Pakistan, where pyrazinamide resistance was significantly lower than rifampicin resistance in all patient groups (p<0·0001). Proportions of pyrazinamide resistance were significantly higher among patients with rifampicin-resistant tuberculosis (compared with patients with rifampicin-susceptible tuberculosis), and among patients previously treated for tuberculosis (compared with those never treated for tuberculosis) in almost all settings.

5015 patients were tested to investigate resistance to fluoroquinolones. Proportions of resistance to individual fluoroquinolones by setting, history of previous treatment, and rifampicin susceptibility are described in table 2 (ofloxacin), table 3 (levofloxacin), table 4 (moxifloxacin), and table 5 (gatifloxacin). Within a particular country, the proportion of all cases with resistance was similar for three drugs (ofloxacin, levofloxacin, and moxifloxacin 0·5 μg/mL). Comparing among countries, the resistance values for all cases ranged from 1·0% to 16·6% (ofloxacin), 0·5% to 12·4% (levofloxacin), and 0·9% to 14·6% (moxifloxacin 0·5 μg/mL). By contrast, the ranges for resistance among all cases for moxifloxacin 2 μg/mL and gatifloxacin were 0·0–5·1% and 0·0–2·5%, respectively, and thus were both lower and less variable.

The proportion of new cases of fluoroquinolone resistance was significantly lower than the proportion of rifampicin resistance cases in all countries except Bangladesh and Pakistan. Higher levels of ofloxacin resistance were found in all settings among rifampicin-resistant tuberculosis cases compared with rifampicin-susceptible tuberculosis cases, but this association was not significant in any setting when compared with the other fluoroquinolones. Finally, we observed higher resistance in retreatment than in new cases, but this finding was statistically significant in only half or fewer of the six settings.

Of the 303 isolates that were resistant to ofloxacin in this study, drug-susceptibility testing results for all other fluoroquinolones were available for 282 (93%). Proportions of cross resistance among ofloxacin-resistant isolates are presented in table 6 and were high for levofloxacin (87%), and moxifloxacin when tested at 0·5 μg/mL (72%). However, cross resistance remained very low between ofloxacin and moxifloxacin (when tested at 2·0 μg/mL; 7%), and gatifloxacin (2%).

## Discussion

In this multicountry survey, we present the first population-based data for the prevalence of resistance to pyrazinamide and fluoroquinolones among patients with tuberculosis in high-burden countries. In routine surveillance and patient management, testing for resistance to these drugs is restricted to particular patient groups, such as those with rifampicin resistance or a history of previous tuberculosis treatment. This dataset therefore provides essential insight into background levels of resistance and the feasibility of introducing new tuberculosis treatment regimens and strategies for drug-susceptibility testing in these settings.

Pyrazinamide is a crucial component of the most commonly used short-course regimen for the treatment of tuberculosis recommended by WHO worldwide,^8^ and also of second-line regimens for the treatment of multidrug-resistant tuberculosis.^11^ In the countries investigated, our study showed no significant difference in the overall levels of resistance to pyrazinamide and rifampicin, with the only exception being Pakistan where pyrazinamide resistance was significantly lower than rifampicin resistance. Additionally, pyrazinamide resistance was significantly associated with rifampicin resistance in all settings, confirming that the vast majority of the burden of pyrazinamide resistance is among patients with rifampicin resistance who can be rapidly identified using existing molecular tests. Nonetheless, for a substantial proportion of patients with rifampicin-resistant tuberculosis (19–63%, based on the findings of this study; table 1), pyrazinamide could still be effective. These findings have two important implications. First, rifampicin-sparing regimens that include pyrazinamide might not be more effective than the current first-line regimen for the treatment of tuberculosis at the population level, given that levels of resistance to rifampicin and pyrazinamide are similar. Second, it is crucial to rapidly distinguish between patients who could benefit from a pyrazinamide-containing regimen and those for whom inclusion of pyrazinamide would not be effective. This distinction requires rapid molecular tests for the diagnosis of pyrazinamide resistance, which do not currently exist.^26^

As with pyrazinamide, levels of resistance to ofloxacin were significantly associated with rifampicin resistance in all settings. Levels of resistance to ofloxacin, levofloxacin, and moxifloxacin at 0·5 μg/mL were similar in all settings and significantly lower than those of rifampicin resistance in Azerbaijan and Belarus (despite the high prevalence of multidrug-resistant tuberculosis in both settings). By contrast, in Pakistan (and to a lesser extent in Bangladesh) resistance to fluoroquinolones was higher than rifampicin resistance, an alarming finding. This finding probably results from the extensive use of fluoroquinolones in many parts of Asia for the treatment of not only tuberculosis but also pneumonia and uncomplicated respiratory-tract infections generally.^27^ In eastern Europe and South Africa, fluoroquinolone resistance is mostly confined to patients with rifampicin resistance, reflecting use of this class of antibiotics for tuberculosis treatment only as second-line therapy. In South Africa, although fluoroquinolones are used for the treatment of pneumonia, this use is primarily in the private health sector which is often not accessible to patients with tuberculosis.

Resistance to the latest generation of fluoroquinolones (moxifloxacin and gatifloxacin at 2·0 μg/mL) was extremely low in all settings, even among patients with rifampicin resistance. This finding can be partly explained by the still-infrequent use of fourth-generation fluoroquinolones in most countries. However, it could also represent an underestimation of the real problem in view of the poor understanding of the association between the critical concentration of susceptibility testing of some fluoroquinolones in the laboratory and patient clinical outcomes. Recent data suggest that the breakpoint of 2·0 μg/mL in liquid media might in fact be too high.^28^, ^29^

The finding of extensive cross resistance between ofloxacin, levofloxacin, and moxifloxacin at 0·5 μg/mL (table 6) was expected and in line with the results of genetic studies.^30^ Importantly, cross resistance is still very limited between ofloxacin at 2·0 μg/mL and either moxifloxacin at 2·0 μg/mL or gatifloxacin, but this may partly be a consequence of the excessively high breakpoints as already discussed. This finding supports the current recommendations of using moxifloxacin or gatifloxacin in the treatment of multidrug-resistant tuberculosis.^11^

Our study has two main limitations. The first is related to its retrospective nature. Surveys were designed to investigate proportions of multidrug-resistant tuberculosis, not proportions of resistance to pyrazinamide or fluoroquinolones. As a consequence, when very low levels of resistance were detected (particularly to moxifloxacin at 2·0 μg/mL and gatifloxacin), the estimates have wide confidence intervals (Table 4, Table 5). Although higher levels of resistance to most drugs were evident among rifampicin-resistant and previously treated cases, statistically significant differences could not be detected in all settings due to insufficient power. Additionally, a proportion—generally below 10%, but 29% in Bangladesh—of the original surveys strains could not be regrown. Imputation of missing values was performed to address this.^14^

A second limitation is related to the laboratory component. Although critical concentrations recommended by WHO^25^ were used for phenotypic testing, recent evidence suggests that some of these thresholds might not be ideal.^20^, ^28^, ^29^, ^30^, ^31^ In particular, the low detected levels of resistance to moxifloxacin and gatifloxacin could be a consequence of excessively high breakpoints. To estimate levels of resistance to pyrazinamide, we largely relied on *pncA* mutations to avoid problems related to the suboptimal reproducibility of phenotypic testing and uncertainties around the most appropriate critical concentration.^20^, ^25^, ^31^ It is expected that *pncA* mutations identify around 85–90% of all existing resistance to pyrazinamide^32^, ^33^ and consequently our results may slightly underestimate the true burden of resistance. Additionally, although only methods recognised by WHO were used and all laboratories passed proficiency testing before starting the project, a level of variability in the results between laboratories can be expected.

Our findings show that the presence of rifampicin resistance, which can be easily identified due to the wide availability of new rapid molecular technologies, should draw attention to the possibility of the simultaneous presence of resistance to pyrazinamide and, in some settings, the earlier-generation fluoroquinolones. At the same time, resistance to the latest generation of fluoroquinolones at the clinical breakpoint is still uncommon. These findings are crucial for the design of standard regimens in different settings, guidance to regimen developers and diagnostic manufacturers, and the introduction of existing regimens for the treatment of drug-resistant tuberculosis (eg, such as shorter regimens).^11^ Choices about prioritisation and introduction of new regimens and algorithms for drug-susceptibility testing must take these data into consideration, and surveillance approaches need to be re-thought so that better data are available to understand levels of resistance to pyrazinamide and fluoroquinolones in different epidemiological settings and patient groups. Without this information, the risk of introducing ineffective regimens that are not curative and might amplify development of drug resistance, including to new agents, remains high. Progress towards drug-susceptibility testing in all cases and rapid development of sequencing technologies for detection of mutations expressing resistance to as many drugs as possible is crucial to optimise treatment and prevent the creation of additional drug resistance.

## Figures and Tables

**Table 1:** Results of sequencing of the *pncA* gene (associated with pyrazinamide resistance)

	**Azerbaijan**	**Bangladesh**	**Belarus (Minsk city)**	**Pakistan**	**South Africa (Gauteng)**	**South Africa (KwaZulu Natal)**
New tuberculosis cases	530, 10·2% (7·6–12·8)	751, 2·6% (1·4–3·8)	144, 30·0% (22·6–37·3)	1299, 2·1% (1·3–2·9)	648, 2·8% (1·5–4·1)	444, 2·0% (0·8–3·3)
Previously treated tuberculosis cases	218, 17·9% (12·7–23·0)	203, 13·8% (8·8–18·8)	57, 69·9% (58·4–81·4)	201, 8·9% (5·1–12·8)	145, 4·7% (1·5–7·8)	128, 10·5% (5·1–15·8)
All tuberculosis cases	748, 12·6% (10·1–15·0)	955, 5·1% (3·4–6·8)	201, 42·1% (35·4–48·8)	1500, 3·0% (2·0–4·0)	877, 3·1% (1·9–4·4)	691, 3·9% (2·4–5·4)
Rifampicin susceptible	619, 2·2% (1·1–3·4)	892, 2·5% (1·3–3·6)	103, 4·2% (0·1–8·3)	1397, 0·5% (0·1–0·8)	838, 1·3% (0·4–2·2)	657, 1·3% (0·4–2·3)
Rifampicin resistant	129, 59·9% (51·0–68·9)	63, 36·7% (25·9–47·4)	98, 81·3% (73·7–88·9)	103, 39·5% (30·1–48·9)	39, 39·1% (22·9–55·3)	34, 49·1% (32·7–65·5)
Resistance in rifampicin resistant *vs* rifampicin susceptible	<0·0001	<0·0001	<0·0001	<0·0001	<0·0001	<0·0001
Resistance in newly *vs* previously treated	0·004	<0·0001	<0·0001	<0·0001	0·169	<0·0001

Data are number tested, % resistant (95% CI) or p value.

**Table 2:** Ofloxacin 2·0 μg/mL susceptibility testing results

	**Azerbaijan**	**Bangladesh**	**Belarus (Minsk city)**	**Pakistan**	**South Africa (Gauteng)**	**South Africa (Kwazulu Natal)**
New tuberculosis cases	528, 3·4% (1·9–5·0)	736, 4·4% (2·6–6·2)	141, 7·0% (2·7–11·3)	1301, 11·2% (7·8–14·7)	716, 1·0% (0·1–1·8)	437, 1·0% (0·0–2·1)
Previously treated tuberculosis cases	220, 8·6% (5·0–12·3)	196, 9·2% (5·1–13·3)	55, 38·8% (26·2–51·4)	202, 15·1% (10·0–20·3)	153, 0·8% (0·0–2·5)	125, 2·0% (0·0–4·2)
All tuberculosis cases	748, 5·0% (3·4–6·6)	933, 5·5% (3·7–7·3)	196, 16·6% (11·4–21·9)	1503, 11·8% (8·7–14·9)	960, 1·0% (0·0–1·8)	675, 1·3% (0·4–2·2)
Rifampicin susceptible	618, 0·7% (0·0–1·3)	873, 4·6% (3·0–6·2)	99, 2·9% (0·0–6·3)	1401, 11·1% (7·8–14·3)	919, 0·4% (0·0–0·9)	637, 0·2% (0·0–0·7)
Rifampicin resistant	130, 25·0% (17·6–32·4)	60, 16·0% (6·3–25·7)	97, 30·7% (21·5–40·0)	102, 21·8% (13·1–30·5)	41, 12·3% (1·5–23·2)	33, 18·3% (5·7–31·0)
Resistance in rifampicin resistant *vs* rifampicin susceptible	<0·0001	0·001	<0·0001	0·009	<0·0001	<0·0001
Resistance in newly *vs* previously treated	0·004	0·014	<0·0001	0·186	0·868	0·383

Data are number tested, % resistant (95% CI) or p value.

**Table 3:** Levofloxacin 1·5 μg/mL susceptibility testing results

	**Azerbaijan**	**Bangladesh**	**Belarus (Minsk city)**	**Pakistan**	**South Africa (Gauteng)**	**South Africa (KwaZulu Natal)**
New tuberculosis cases	527, 2·2% (1·2–4·0)	729, 3·3% (1·7–5·0)	141, 4·7% (1·2–8·1)	1299, 10·1% (6·7–13·4)	705, 0·5% (0·0–1·1)	419, 0·5% (0·0–1·3)
Previously treated tuberculosis cases	220, 6·9% (3·6–10·3)	192, 5·4% (2·6–8·3)	55, 30·3% (18·7–41·9)	201, 14·9% (9·8–19·9)	151, 0·5% (0·0–1·2)	121, 0·9% (0·0–2·6)
All tuberculosis cases	747, 3·9% (2·5–5·3)	921, 3·8% (2·3–5·3)	196, 12·4% (7·8–17·0)	1500, 10·7% (7·7–13·7)	945, 0·5% (0·0–1·1)	650, 0·6% (0·0–1·3)
Rifampicin susceptible	618, 0·5% (0·0–1·1)	866, 3·6% (2·1–5·2)	99, 2·8% (0·0–6·0)	1401, 10·3% (7·1–13·5)	806, 0·2% (0·0–0·9)	620, 0·3% (0·0–1·4)
Rifampicin resistant	129, 19·4% (12·5–26·3)	56, 5·5% (0·0–12·0)	97, 22·4% (14·2–30·6)	99, 21·8% (13·1–30·5)	39, 7·0% (0·0–15·1)	30, 7·9% (0·0–17·5)
Resistance in rifampicin resistant *vs* rifampicin susceptible	<0·0001	0·537	0·002	0·151	0·040	0·040
Resistance in newly *vs* previously treated	0·007	0·156	<0·0001	0·100	0·280	0·661

Data are number tested, % resistant (95% CI) or p value.

**Table 4:** Moxifloxacin 0·5 μg/mL and 2·0 μg/mL susceptibility testing results

	**Azerbaijan**	**Bangladesh**	**Belarus (Minsk city)**	**Pakistan**	**South Africa (Gauteng)**	**South Africa (KwaZulu Natal)**
**0·5 μg/mL**						
New tuberculosis cases	528, 2·2% (0·9–3·4)	736, 3·8% (2·2–5·4)	141, 6·3% (2·2–10·4)	1301, 7·5% (5·9–9·1)	709, 0·8% (0·0–1·6)	421, 0·6% (0·0–1·4)
Previously treated tuberculosis cases	220, 7·0% (3·6–10·4)	196, 7·0% (3·7–10·4)	55, 33·5% (21·7–45·4)	202, 12·1% (7·3–16·8)	152, 0·9% (0·0–2·8)	123, 2·1% (0·0–4·3)
All tuberculosis cases	748, 3·6% (2·3–5·0)	933, 4·5% (2·9–6·1)	196, 14·6% (9·6–19·5)	1503, 8·1% (6·7–9·6)	951, 0·9% (0·0–1·7)	654, 1·0% (0·2–1·7)
Rifampicin susceptible	618, 0·5% (0·0–1·1)	873, 3·9% (2·4–5·3)	99, 2·7% (0·0–5·9)	1401, 7·7% (6·1–9·3)	910, 0·5% (0·0–1·1)	621, 0·3% (0·0–0·8)
Rifampicin resistant	130, 17·9% (11·2–24·5)	60, 12·2% (3·7–20·7)	97, 26·8% (18·0–35·7)	102, 13·8% (6·3–21·4)	41, 8·4% (0·0–18·4)	33, 12·2% (2·2–22·2)
Resistance in rifampicin resistant *vs* rifampicin susceptible	<0·0001	0·007	<0·0001	0·075	<0·0001	<0·0001
Resistance in newly *vs* previously treated	0·002	0·044	<0·0001	0·053	0·919	0·174
**2·0 μg/mL**						
New tuberculosis cases	528, 0·4% (0·0–1·0)	732, 0·4% (0·0–1·0)	141, 1·2% (0·0–3·3)	1299, 0·4% (0·0–0·8)	707, 0·5% (0·0–1·4)	420, 0·0% (0·0–11·2)
Previously treated tuberculosis cases	220, 0·5% (0·0–1·5)	192, 1·4% (0·0–3·1)	55, 14·0% (4·5–23·5)	202, 1·7% (0·0–4·0)	151, 0·7% (0·0–2·0)	121, 0·0% (0·0–3·0)
All tuberculosis cases	748, 0·5% (0·0–1·0)	925, 0·7% (0·0–1·3)	196, 5·1% (1·7–8·6)	1501, 0·5% (0·0–1·1)	948, 0·5% (0·0–1·1)	651, 0·0% (0·0–0·6)
Rifampicin susceptible	618, 0·9% (0·0–3·7)	869, 0·4% (0·0–0·9)	99, 4·5% (0·0–13·9)	1401, 0·5% (0·0–1·0)	908, 0·3% (0·0–0·7)	620, 0·0% (0·0–0·6)
Rifampicin resistant	130, 2·0% (0·0–5·6)	56, 3·2% (0·0–8·3)	97, 9·2% (3·1–15·3)	100, 1·4% (0·0–4·3)	40, 3·8% (0·0–10·8)	31, 0·0% (0·0–11·2)
Resistance in rifampicin resistant *vs* rifampicin susceptible	0·410	0·051	0·270	0·316	0·008	..
Resistance in newly *vs* previously treated	0·926	0·247	0·007	0·132	0·653	..

Data are number tested, % resistant (95% CI) or p value.

**Table 5:** Gatifloxacin 2·0 μg/mL susceptibility testing results

	**Azerbaijan**	**Bangladesh**	**Belarus (Minsk city)**	**Pakistan**	**South Africa (Gauteng)**	**South Africa (KwaZulu Natal)**
New tuberculosis cases	528, 0·6% (0·0–1·4)	729, 0·0% (0·0–0·5)	141, 1·2% (0·0–3·2)	1299, 0·0% (0·0–0·3)	705, 0·0% (0·0–0·5)	419, 0·0% (0·0–0·9)
Previously treated tuberculosis cases	220, 0·5% (0·0–1·5)	192, 0·0% (0·0–1·9)	55, 5·4% (0·0–12·4)	201, 0·0% (0·0–1·8)	151, 0·0% (0·0–2·4)	121, 0·0% (0·0–3·0)
All tuberculosis cases	748, 0·6% (0·0–1·2)	922, 0·0% (0·0–0·4)	196, 2·5% (0·0–5·2)	1500, 0·0% (0·0–0·2)	945, 0·0% (0·0–0·4)	650, 0·0% (0·0–0·6)
Rifampicin susceptible	618, 1·9% (0·0–6·9)	866, 0·0% (0·0–0·4)	99, 3·0% (0·0–7·2)	1401, 0·0% (0·0–0·3)	906, 0·0% (0·0–0·4)	620, 0·0% (0·0–0·6)
Rifampicin resistant	130, 3·3% (0·1–6·5)	56, 0·0% (0·0–6·4)	97, 4·0% (0·0–8·5)	99, 0·0% (0·0–3·7)	39, 0·0% (0·0–9·0)	30, 0·0% (0·0–11·6)
Resistance in rifampicin resistant *vs* rifampicin susceptible	0·310	..	0·270	..	..	..
Resistance in newly *vs* previously treated	0·818	..	0·143	..	..	..

Data are number tested, % resistant (95% CI) or p value.

**Table 6:** Cross resistance among fluoroquinolones

	**No resistant strains**	**No susceptible strains**	**% resistant strains**
Ofloxacin (2·0 μg/mL)	282	0	100%
Levofloxacin (1·5 μg/mL)	245	37	87%
Moxifloxacin (0·5 μg/mL)	203	79	72%
Moxifloxacin (2·0 μg/mL)	19	263	7%
Gatifloxacin (2·0 μg/mL)	7	275	2%
