# Supplementary material for: Population-based resistance of Mycobacterium tuberculosis isolates to pyrazinamide and fluoroquinolones: results from a multicountry surveillance project
Source: Lancet Infect Dis. 2016 Oct;16(10):1185–92. doi: 10.1016/S1473-3099(16)30190-6 (PMC5030278; doi:10.1016/S1473-3099(16)30190-6)
Supplement: Supplementary appendix [file mmc1.pdf]

THE LANCET Infectious Diseases

Supplementary webappendix

This webappendix formed part of the original submission and has been peer reviewed. We post it as supplied by the authors.

Supplement to: Zignol M, Dean AS, Alikhanova N, et al. Population-based resistance of *Mycobacterium tuberculosis* isolates to pyrazinamide and fluoroquinolones: results from a multicountry surveillance project. *Lancet Infect Dis* 2016; published online July 7. http://dx.doi.org/10.1016/S1473-3099(16)30190-6.

Appendix

Profile of countries included in the study. Data already presented elsewhere.

	Azerbaijan [1]	Bangladesh [1]	Belarus [1]	Pakistan [1]	South Africa [1]	
TB incidence number (thousands) in 2014	7.4 (6.5-8.3)	360 (320-410)	5.5 (4.7-6.7)	500 (370-650)	450 (400-510)	
TB incidence rate (per 100,000) in 2014	77 (68-86)	227 (200-256)	58 (50-67)	270 (201-350)	834 (737-936)	
Total TB cases notified in 2014	7,539	196,797	4,274	316,577	318,193	
Proportion of HIV positive TB patients in 2014	2%	4%	6%	<1%	61%	
Site and year of drug resistance survey	Nationwide, 2013 [15]	Nationwide, 2013 [16]	Minsk, 2010 [17]	Nationwide, 2013 [18]	Gauteng, 2014 [19]	Kwazulu Natal, 2014 [19]
New TB cases tested and<br>% rifampicin resistance	549<br>13.3 (10.6-16.4)	1,050<br>1.6 (0.9-2.9)	156<br>36.5 (28.9-44.2)	1,380<br>4.2 (3.2-5.3)	837<br>3.6 (2.1-5.2)	499<br>3.5 (1.6-5.5)
Previoulsy treated TB cases tested and<br>% rifampicin resistance	240<br>28.3 (22.7-34.5)	291<br>28.9 (23.9-34.4)	68<br>77.9 (67.8-88.1)	212<br>19.8 (15.1-22.5)	175<br>9.3 (4.8-13.8)	141<br>8.8 (3.0-14.6)
All TB cases tested and<br>% rifampicin resistance	789<br>17.9 (15.3-20.7)	1344*<br>7.3 (5.2-10.1)	224<br>49.1 (42.5-55.7)	1,592<br>6.3 (5.3-7.5)	1,123*<br>4.8 (3.4-6.8)	784*<br>4.9 (3.2-7.5)

Ranges represent uncertainty intervals. * Total number includes patients with history of treatment unknown.

Characteristics of patients enrolled in the study. Data already presented elsewhere.

	Azerbaijan [15]	Bangladesh [16]	Belarus (Minsk city) [17]	Pakistan [18]	South Africa (Gauteng) [19]	South Africa (Kwazulu Natal) [19]
Total	789	1344	224	1592	1,123	784
- male	587, 74.4%	949, 70.6%	157, 70.1%	884, 55.5%	691, 61.5%	483, 61.6%
- female	202, 25.6%	395, 29.4%	67, 29.9%	708, 44.5%	401, 35.7%	291, 37.1%
- unknown					31, 2.8%	10, 1.3%
Age group, years						
- up to 24	178, 22.6%	290, 21.6%	20, 8.9%	483, 30.3%	95, 8.5%	108, 13.8%
- 25-34	191, 24.2%	318, 23.7%	54, 24.1%	333, 20.9%	369, 32.9%	285, 36.4%
- 35-44	184, 23.3%	216, 16.1%	52, 23.2%	232, 14.6%	370, 33.0%	206, 26.3%
- 45-54	133, 16.9%	189, 14.1%	61, 27.2%	234, 14.7%	187, 16.7%	100, 12.8%
- 55-64	72, 9,1%	175, 13.0%	22, 9.8%	171, 10.7%	66, 5.9%	47, 6.0%
- over 65	31, 3.9%	152, 11.3%	15, 6.7%	133, 8.4%	17, 1.5%	14, 1.8%
- unknown		4, 0.3%		6, 0.4%	19, 1.7%	24, 3.1%
Treatment history						
- new	549, 69.6%	1050, 78,1%	156, 69.6%	1380, 86.7%	837, 74.5%	499, 63.6%
- previously treated	240, 30.4%	291, 21.7%	68, 30.4%	212, 13.3%	175, 15.6%	141, 18.0%
- unknown		3, 0.2%			111, 9.9%	144, 18.4%

Data are absolute numbers and proportions.
